# Nelligan’s Treatment of Eruptive Diseases

**Published:** 1850-09

**Authors:** J. Moore Nelligan


### ARTICLE VI.

*Suggestions on the Treatment of Eruptive Diseases of the Skin.*—By J. MOORE NELLIGAN, M. D., M. R. I. A., &c.

[Speaking of the chronic squamous diseases of the skin, and more particularly of psoriasis, Dr. Nelligan gives the following practical hints:—

Donavan's solution of the Hydriodate of Arsenic and Mercury has been extensively used in the treatment of this class of cutaneous eruptions. I must, however, confess that I am not partial to the employment of any mercurial preparation in their treatment, when they affect the body generally. The only local form of scaly disease in which I have found a mercurial useful, is in pityriasis, and in it I find the yellow Iodide of Mercury productive of the best results.

The Iodide of Arsenic has also been found a remedy of decided efficacy in the treatment of these affections; it was first generally employed by Dr. A. T. Thompson. My own experience leads me to place more reliance upon it than on any other preparation of Arsenic; but though I found it alone capable of curing many cases of psoriasis and lepra, I consider that its beneficial action is much augmented by combining it with Iodide of Potassium and Iodine.

On the importance of a milk diet, my experience leads me to the conclusion that an adherence to it is of the utmost consequence in cutaneous eruptions, except where they occur in cachetic or broken down constitutions, or are attended with ulcers, or profuse and weakening discharges.

Tepid baths of *fresh* water, twice or three times weekly, will be found an adjunct of much importance in the treatment of scaly eruptions; they tend to restore the natural secretions, and prevent the accumulation of scales. They are especially useful when much local irritation exists. I have tried various local applications, but my experience leads me to prefer the tepid bath.

[The mixture of Arsenic and Hydriodate of Potash alluded to, is made according to the following formula:—

℞. Liq. Arsenicalis, ʒij.

Syrup, Simp., ʒij.

Aquæ Dist., ʒss.

Potass. Iodide, ʒss.

Of this a dessert-spoonful is given three three times a-day in water. To this mixture, in inveterate cases, six grains of Iodine are added.]

