# Important Erratum

**Published:** 1851-03

**Authors:** 


### ARTICLE IX.

### IMPORTANT ERRATUM.

Dr. B. Woodward, of Sharon, Illinois, calls our attention to the error in the formula, at the close of Dr. Nelligau's article on the treatment of Diseases of the Skin, published in No. 4, of this volume, page 243. The water should have been put f. ℥ ss instead of f. ʒ ss.

We should think the dose of arsenic full large, even after this correction, as the dose given wonld contain about one-fourth of a grain of arsenic. Better begin with a tea-spoonful than a desert-spoonful.

